# Online Pharmacies Selling Prescription Drugs: Systematic Review

**DOI:** 10.3390/pharmacy10020042

**Published:** 2022-04-01

**Authors:** Chiau Soon Long, Harshily Kumaran, Khang Wen Goh, Faizah Safina Bakrin, Long Chiau Ming, Inayat Ur Rehman, Jagjit Singh Dhaliwal, Muhammad Abdul Hadi, Yee Wai Sim, Ching Siang Tan

**Affiliations:** 1 Faculty of Computing and Engineering, Quest International University, Ipoh 30250, Malaysia; chiausoon.long@qiu.edu.my; 2 School of Pharmacy, KPJ Healthcare University College, Nilai 71800, Malaysia; harshilykumaran@gmail.com (H.K.); ucn.faizah_safina@kpjuc.edu.my (F.S.B.); 3 Faculty of Data Science and Information Technology, INTI International University, Nilai 71800, Malaysia; khangwen.goh@newinti.edu.my; 4 PAP Rashidah Sa’adatul Bolkiah Institute of Health Sciences, Universiti Brunei Darussalam, Gadong BE1410, Brunei; longchiauming@gmail.com (L.C.M.); jagjit.dhaliwal@ubd.edu.bn (J.S.D.); 5 Department of Pharmacy, Abdul Wali Khan University Mardan, Mardan 23200, Pakistan; inayat.rehman@awkum.edu.pk; 6 Department of Clinical Pharmacy and Practice, College of Pharmacy, QU Health, Qatar University, Doha P.O. Box 2713, Qatar; mabdulhadi@qu.edu.qa

**Keywords:** internet pharmacy, prescribed medication, medication safety, patient safety, counterfeit medicine

## Abstract

**Introduction**: The patronage of online pharmacies is rapidly growing, driven by the convenience and cheaper costs of purchasing prescription drugs electronically, especially under the lockdown situation. However, there are issues regarding the quality of the prescription drugs sold online and the legitimacy of online pharmacies. The use of prescription drugs without the supervision of a licensed health care practitioner may potentially harm consumers. **Objectives**: This systematic review was conducted to improve the body of knowledge on three main aspects of online pharmacies: (1) type and characteristics of the online pharmacies selling drugs; (2) the quality of pharmaceutical drugs purchased online; and (3) the characteristics of consumers of online pharmacies. **Methods**: Based on a pre-defined search strategy, PubMed and Scopus were utilised to search articles written in the English language published between January 2009 and February 2020. Studies focusing on the sale of prescription drugs were included. The terms used for the literature search were “online pharmacy”, “internet pharmacy”, “e-pharmacy”, “prescription”, “quality”, “medication safety”, and “counterfeit medicine”. These terms were used alone and in combination with Boolean operators. The institutional webpages including the World Health Organization (WHO) and the United States Food and Drug Administration (USFDA) were also examined for any additional studies. No methodological limitations in terms of study design were applied. A standardised data collection form was used to compile the data. **Results**: Based on the inclusion and exclusion criteria, a total of 46 articles were eligible and included in the final analysis. There were 27 articles on types and characteristic of online pharmacies, 13 articles on the quality of prescription drugs sold from online pharmacies, and 11 articles on consumers purchasing prescription drugs from online pharmacies. Readers should note that five articles discussed both the types and characteristics of online pharmacies, and the quality of the drugs sold from the outlets. The response rate (products received out of the number of orders) ranged from 20% to 100%, whereas the proportion of consumers buying prescription drugs online ranged from 2.3% to 13%. Reasons for online purchase of prescription drugs include the difficulty of obtaining a prescription for certain medications such as opioid analgesics, cheaper cost, since the costs associated with seeing a physician to obtain a prescription are reduced, and the need to obtain drugs such as opioid analgesics and benzodiazepine for misuse. **Conclusions**: Almost half of the online pharmacies are not properly regulated and fraudulent issues were uncovered. To address this issue, stricter regulation by World Health Organization and implementation should be carried out together with frequent monitoring of the licensure system and pharmacy verification on every online pharmacy, this would reduce the number of illegal or illegitimate online pharmacy.

## 1. Introduction 

The number of online pharmacies has been rising over the years. The internet has allowed patients and consumers to buy a range of medicinal products from online pharmacies at a comparatively lower cost than from retail pharmacies. However, some illicit websites based in many countries have been posing as legal pharmacies and are taking advantage of the internet [1] to sell adulterated drugs and low-quality medications [2]. In addition, some medicines are sold illegally without the need for prescriptions [2]. This phenomenon should be a cause for concern since low quality medicines can be unsafe. Furthermore, unsupervised and self-medication use of drugs may expose consumers to harmful adverse effects and increase the risk of morbidity and mortality [3].

The availability of and accessibility to substandard and counterfeit drug products may differ among countries. Countries with good pharmaceutical regulatory and quality assurance systems generally have a lower prevalence of fake or counterfeit products compared to countries with poor regulatory systems. For instance, the availability of counterfeit drugs in Japan and South Korea is low, but it is high in countries such as China and India [4,5]. A large number of counterfeit and fake medicines have been reported to be available in the markets [6]. India has been reported to have the highest prevalence of manufactured counterfeit drugs, which is up to 35%, followed by Nigeria (23.1%) and Pakistan (13.3%) [4]. Nevertheless, counterfeit drugs can still be available from the black market in countries with advanced pharmaceutical regulatory systems [7]. 

The World Health Organization (WHO) has identified several factors influencing the availability of counterfeit drugs in the black market. These factors include poor national regulation for drug distribution and manufacturing, limited enforcement of drug legislation, poor penal sanctions for violations of drugs legislation and poor regulation among the exporting countries [4]. Other important factors include the complex transactions involving many intermediaries, the high demand and cost for curative and prophylactic drugs, and inefficient collaboration among stakeholders [4]. 

There is increasing patronage of online pharmacies and the reasons include the convenience of accessing pharmaceutical drugs from these online platforms and their comparatively lower cost than products obtained from physical pharmacies [8]. Nevertheless, issues regarding the quality of online prescription drugs have been a major public health concern. In addition, the legitimacy of online pharmacies selling pharmaceutical drugs may be questionable [9]. Most importantly, there are concerns regarding the safety of consumers since inappropriate use of prescription drugs may cause potential harm [10,11]. Therefore, the present study aimed to conduct a systematic review about online pharmacies by focusing on three main areas: (1) type and characteristic of online pharmacies; (2) quality of pharmaceutical drugs purchased online; and (3) characteristics of consumers purchasing pharmaceutical drugs from online pharmacies. 

## 2. Methodology

### 2.1. Study Design 

This systematic review was designed based on the previous work published by Orizio et al. [9]. Original studies published between January 2009 and February 2020 were obtained, compiled, and summarised for this review. 

### 2.2. Search Strategy

Two electronic databases, namely PubMed and Scopus, were employed to search for the relevant articles. Related studies were accessed from the institutional websites of the WHO and Food Drug Administration (FDA). The search terms used to identify the studies included “online pharmacy”, “internet pharmacy”, “e-pharmacy”, “prescription”, “quality”, “medication safety”, and “counterfeit medicine”. These terms were either used alone or in combination with Boolean operators. This systematic review was performed and reported as recommended by the Preferred Reporting Items for Systematic Reviews and Meta-Analyses (PRISMA) [12].

### 2.3. Inclusion and Exclusion Criteria 

The studies included in this review had to fulfil the following criteria: (1) papers published between January 2009 to February 2020; (2) full articles that were written in the English language and; (3) scientific research articles published in peer-reviewed journals. Studies were excluded if the emphasis was on over-the-counter medicines, complementary medicines, herbal remedies, supplements, abused drugs, or social media marketing of pharmaceutical products. Additionally, research notes, letters to the editor, review articles, case reports, and conference papers were not included in this review. 

### 2.4. Data Collection

All search results were exported to a designated EndNote library. Duplicate papers were removed and one author screened the titles and abstracts of all the studies. After that, the full text of relevant titles was downloaded and they were independently assessed for eligibility by two authors. Data were extracted by one author and checked by another author for accuracy. 

The selected articles were divided into:

- Types and Characteristics of Online Pharmacies

The following details were recorded and summarised in this review: the number of online pharmacies analysed/studied in each article, year of publication, availability of a physician’s assistance or online medical consultation, dispensing of pharmaceutical drugs with or without a prescription, disclosure of contact details, geographical locations, delivery conditions, types of available medicines, availability of drug information, drug and other costs, sales promotion strategies, duration to access the websites, availability of privacy and disclaimer statements, date of last website update, and presence of professional certifications.

- Online Pharmacy Ordering Service

The data collected for this aspect include the information regarding the type of drug ordered, the response rate, the quality of the process, and the type of drug purchased. The information on the characteristics of the drug purchasing process were recorded. This information includes the requirement of prescription, online questionnaire, money transactions, and subsequent advertising regarding drug quality, information on packaging and instructions, and chemical composition.

- Characteristics of Consumers Purchasing Drugs from Online Pharmacies.

The data collected include commonly requested drugs, the main reasons for online procurement of pharmaceutical drugs, the significance of the location of online pharmacies, and the perceived risks associated with the practice. 

## 3. Results

**Figure 1:**
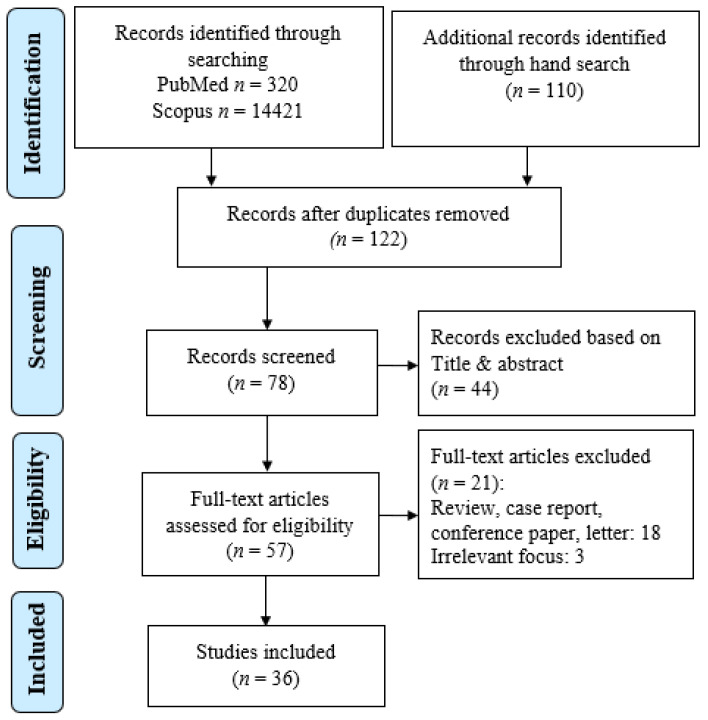
PRISMA flowchart for the included studies (*n* denotes the number of article/study).

The systematic search of articles resulted in the compilation of 27 articles that described the types and characteristics of online pharmacies, 13 articles that focused on the quality of drugs sold from online pharmacies and 11 articles that reported the characteristics of consumers purchasing drugs from online pharmacies. In addition, five articles discussed both the types and characteristics of online pharmacies and the quality of drugs sold from the platforms. The results for the article search are outlined in Figure 1. 

### 3.1. Types and Characteristic of Online Pharmacies

The types and characteristics of online pharmacies included in this systematic review and its findings are tabulated in Table 1.

### 3.2. Quality of Drugs Purchased

A total of eight studies examined the type of drugs that are procured online without prescriptions and assessed their quality based on various aspects (Table 2). Examples of the drugs included drugs to treat erectile dysfunction, proton pump inhibitors, non-steroidal anti-inflammatory drugs, antihyperlipidemic agents, antibiotics, antidepressants, and antiretroviral drugs. 

In this study, response rate was assessed by comparing the number of orders to the number of products received. Our findings showed that response rates varied among the studies, ranging from 20% [30] to 100% [29,36]. Some studies did not report the response rates. Various approaches were used in the study to determine the quality of online purchased drugs. These approaches included the evaluation of drugs’ information, chemical content and the packaging upon receipt of the products.

### 3.3. Characteristics of Consumer Buying from Online Pharmacies

Table 3 shows the characteristics of consumers who patronise online pharmacies (internet and websites). The information includes the consumers’ profile, the location (i.e., country) in which the study was conducted and the percentage of consumers buying the products. Based on the reviewed studies, the characteristics of consumers who patronise online pharmacies included young men, adolescents, college and university students, drug abusers, street workers, healthy adults and white females. Most of the reviewed studies were cross-sectional surveys. 

Overall, the percentage of respondents who admitted to buying prescription drugs online ranged from 1% to 32%. One of the important factors influencing respondents’ practices towards engaging in online drug purchase was the reluctance of physicians to prescribe certain medications. For instance, opioid analgesics are not easily prescribed for pain due to the drug’s tendency to cause dependence. Other factors associated with online drug procurement include the lack of physician fee and the intention to obtain drugs such as opioid analgesics and benzodiazepine for misuse [30].

## 4. Discussion

### 4.1. Types and Characteristics of Online Pharmacies

#### 4.1.1. Prescription Requirement

The prescriptions submitted to online pharmacies were usually faxed or emailed as a scanned copy. Some online pharmacies request an updated prescription. This review confirmed several findings documented in the literature, emphasising how medicines, including prescription-only drugs, are obtained online with or without prescriptions [13,26,32]. It is of note that some consumers intentionally procure prescription medications online without a prescription, especially from rogue pharmacies [1,46]. Most of the drugs are purchased to manage chronic conditions such as psychiatric and cardiac disorders [27,54]. 

Patients with mental disorders have higher tendencies to engage in self-diagnosis and self-medication [13,18,27,55]. Hence, studies conducted on the sale of psychiatric drugs found that patients with mental illness used online pharmacies to stock in drugs believed to be effective in managing their condition. In addition, even when a prescription is available for the drugs, the consumer might still misuse it by sending a single prescription to multiple online pharmacies [41]. The prescription requirement is an important criterion that should be obeyed. However, it is equally vital for prescribed drugs to be procured. Reusing or misusing prescriptions will increase accessibility to prescription drugs, which may put the consumer at risk. Therefore, strategies should be put in place to prevent multiple uses of one prescription through online pharmacies. On that note, only the original prescription should be accepted by online pharmacies, rather than permitting the use of scanned copy via fax or email. 

#### 4.1.2. Presence of Online Questionnaire

The need for a consumer to complete a questionnaire to be completed in addition to a prescription before online drug purchase has been re-established by some studies [56,57]. According to these authors, the questionnaire is a medium to boosting consumers’ confidence regarding the regulatory standards of an online pharmacy. Contraindications and interactions of medicines may occur in patients if they are not well-informed about the drug before online procurement. For instance, patients may bypass the online instrument or questionnaire through a default process without entering any patient-specific information. These events highlight the importance of questionnaires during online drug purchase. Nevertheless, large amounts of prescription medicines could still be ordered online with unverified patient questionnaires [56,58]. 

Another practice that might heighten patient risk and compromise their safety is the use of pre-selected answers on online questionnaires. Pre-selected questionnaires are easily completed and facilitate rapid approval of drug purchase or treatment without the consumer or patient comprehending the questions. In the same vein, pre-selected answers increase the likelihood of a patient skipping a question unintentionally, leading to a ‘false positive’ outcome. This will eventually compromise patient safety [19,30,59]. 

#### 4.1.3. Contact Details

A large percentage of online pharmacies display their contact details [17,60]. A study revealed that 37% of pharmacies provide a telephone number for costumers’ enquiries [61]. So, it seems appropriate for patients to test any telephone numbers provided for authenticity before they make a purchase. Bessel et al.’s findings revealed that only 35% of their online pharmacies had published the owners’ or the director’s name [62]. In order to combat this problem, it is recommended that each country’s pharmacy board or regulatory system should conduct a proper investigation of the locally available online pharmacies and systematically check their websites through the provided contact details.

#### 4.1.4. Geographical Information

Consumers are able to engage in the personal importation of these drugs from other countries. Nevertheless, they may or may not be legally allowed because most countries have no proper legislation to either allow or prohibit online pharmacies [3]. Legitimate pharmacies are mostly located in Europe and the United States [30]. Based on the results, the most common locations mentioned by almost half of the online pharmacies were the United States, Canada and the United Kingdom. A few respondents stated that online pharmacies requiring valid prescriptions are more likely to reveal their geographical locations than rogue ones [17,57].

#### 4.1.5. Delivery Information

Regarding the websites used for online drug procurement, 42% of them declared the origin and destination of the products [13]. Among the websites publicising such information, 47% of them were in Asia (47%) [30]. However, some products were delivered for free. The average waiting time for the medication delivery was eight days [30]. Another study highlighted that several purchased drugs were shipped from a different place than the one indicated on the website [1]. Lagan et al. mentioned that out of eight purchasing requests, seven were successfully delivered. One of the requests was not delivered because the online transaction was rejected. This was further attributed to credit card blockade as the delivery company was blacklisted [22].

#### 4.1.6. Drugs Offered

The drugs offered were sildenafil, obesity reduction medications, benzodiazepines, painkillers, antidepressants and antibiotics. Some studies focused on specific classes of drugs such as sildenafil [32], and medications to ameliorate erectile dysfunction [29] and psoriasis [33]. The formalities involved in the procurement of prescription and non-prescription drugs are completely different. Hence, consumers should be enlightened about the importance and prudent use of prescription drugs. The online purchase of prescription drugs should be tightened. For instance, a robust system should be implemented where the patients are required to post an authentic prescription to the online pharmacy, specific and detailed patient information, and validation from the doctors prescribing the medication. In addition, it is recommended that prescription drugs sold online should either be strictly limited for legitimate consumers or to be sold only to doctors and pharmacists if they find that consumers are committing fraud or misusing the drugs [41].

#### 4.1.7. Drug Prices

Drug prices were one of the factors contributing to excessive procurement and misuse of online drugs. For example, some online pharmacies offered a lower price per tablet if purchased in larger quantities. This encourages customers to buy and store more medicines, which may lead to drug overuse. Campbell et al. [32] mentioned that internet websites that provided prescription sildenafil, regardless of the requirement for a prescription, had a median per tablet price that was 22% higher than Viagra from area pharmacies [15].

In France, a study reported that the mean price of all medicines was higher than the price benchmarked by the country. However, some of the traditional systemic treatments were cheaper than prices in France [25]. Additionally, a 36% discount was also provided for bulk buying of traditional systemic and topical therapies [25]. These findings reinstate the need for consumers to be properly educated regarding drugs and prices. Consumers should understand that they might be substituting quality and authenticity for cheaper-priced drugs [14,31,63].

#### 4.1.8. Marketing Strategies

Levaggi et al. [23] observed the statements that were often used by websites to promote their products. The strategies include a reduction in prices compared to “brick and mortar” pharmacies, free delivery, loyalty bonuses, and special discounts. Another study reported the competitiveness of marketing strategies, focusing on privacy protection and affordability [23]. The marketing of pharmaceutical products was based on special offers such as reduction in prices, which may encourage drug use without prescription or self-medication. This might increase the risk of drug misuse. Therefore, consumers should be aware of the tricks and strategies used by online pharmacies to increase patronage. It will help consumers to differentiate between the good online pharmacies and those that mainly prioritise their income [24].

#### 4.1.9. Quality of Online Pharmacies

Some professional organisations have developed verification and accreditation systems for internet pharmacy to enhance patient safety [23,29]. These systems are designed to distinguish reliable websites from illegal operations [8,56]. The issue of low user safety is enhanced by poor process traceability, along with websites disappearing and a lack of transparency. More importantly, the extreme volatility of websites remains a serious concern from a public health perspective. According to the National Association of Boards of Pharmacy (NABP), 17% of the sampled websites were insecure [28]. Legal enforcement mechanisms empowered the Department of Justice and FDA to shut down websites that violated the Food, Drug, and Cosmetic Act 1938. The act includes the websites marketing the sale of narrow therapeutic index (NTI) drugs without a prescription. Moreover, the FDA should also support legislative action to prohibit online sales of pharmaceutical products by websites which are not accredited by the NABP Verified Internet Pharmacy Practice Site program [28].

The quality of online pharmacies and web vulnerability was investigated by Kuzma et al. [41]. A random sample of 60 online pharmacies was selected and the N-Stalker Web Application Security Scanner 2009 Free Edition 7.0 was used to execute the study objectives [21]. They found that most online pharmacies do not provide sufficient protection for their consumers, especially in cross-site scripting. The authors suggested that online security could be improved by implementing a multi-phased approach. In addition, it will provide a better level of consumer protection and increased consumers’ trust and profitability. Another study revealed that two-thirds of rogue sites displayed stolen and false certifications seals including companies and intellectual property authority (CIPA), fake logos for organisations such as the Food and Drug Administration and Pharmaceutical Group of the European Union, and financial and shipping companies [1,46]. Some online pharmacies use pictures of doctors, professionals’ profiles, official trademarks, and all sorts of publically available information [3]. These practices make it difficult to distinguish between legitimate and illegitimate internet pharmacies [3].

#### 4.1.10. Time of Existence of the Websites and Privacy Policy

The legality of online pharmacies can be determined based on the time of existence. Most of the online pharmacies operating non-prescription internet pharmacies are characterized by the limited availability of patients’ information and medication safety. They also operate illegally and provide medicines to consumers without a proper prescription. These online pharmacies disappear after a while [3,30].

### 4.2. Quality of Drugs Purchased

#### 4.2.1. Drug Characteristics

Based on the drug characteristics, the majority of the purchased medications were prescription drugs. These drugs are classified under the law as protected drugs and can only be procured with valid prescriptions from physicians. However, some online pharmacies accept photocopied or faxed copies of prescriptions. Such practices may result in drug abuse since the same prescription can be used multiple times. In addition, prescription medicines sold by online pharmacies were used inappropriately because they tend to promote self-medication [2]. Another article that evaluated the purchase of isotretinoin without prescription detected that they were still advertising the drug for sale against the directive of the Medicines and Healthcare products Regulatory Agency [22]. Furthermore, the types of drugs purchased were brand-name drugs, and their quality was sometimes influenced by the manufacturing countries. For instance, some of the drugs made in Ethiopia, China, Thailand, Nigeria, India, Laos and Thailand failed the quality tests [3].

#### 4.2.2. Drug Quality Characteristics

Both the internal and external parts of purchased drugs were physically evaluated to determine their quality. Firstly, in terms of packaging, more than half of the drugs were found to be labelled inappropriately. This includes a complete absence of labelling by the manufacturer or spelling errors in the name of the tablets. Specifically, 84% of the drugs purchased lacked leaflets or outer packaging and had damaged packaging [42]. Other issues included the absence of batch number, expiry date, and essential information such as side effects, contraindications, and drug interactions [64]. Another study found that none of the investigated drugs was packed inside a container nor packaged according to the European Council directive. This might increase the risk of consuming expired drugs, interaction with other medications and adverse reactions such as allergy. Nevertheless, some of the drugs had outer packaging but it is not a guarantee of authenticity. It is suggested that the products should be inspected according to good manufacturing practice. Such monitoring should be conducted either by the national or international competent authorities and regulatory agencies [31,34].

### 4.3. Characteristics of Consumers Who Patronise Online Pharmacies

A survey conducted in the United States on the use of erectile dysfunction medications showed that 12% of consumers purchased them on the internet, including undergraduate students [46]. Likewise, a study conducted in Argentina also reported a similar event, as 2.9% of the sales of phosphodiesterase 5 inhibitor (PDE5I) drugs for erectile dysfunction were sold to healthy young men. An observational study on the purchasing patterns of the same substances found that 11% of consumers reported the use of PDE5Is, while 32% of them obtained their drugs from sources outside the healthcare system. In contrast, a study showed no difference in age between consumers buying drugs online and those not engaging in such a practice. Additionally, no difference was observed in terms of student status [30]. However, the consumers on multiple medications were more likely to patronise online pharmacies compared to those with prescriptions [30].

One study tried to expose the “black box” of drug diversion. Drug diversion refers to the transfer of drugs from a legal to an illegal channel using several sources of information. the Delaware School Survey, several Miami street studies, and two qualitative studies [45]. They concluded that the internet is indeed a place for prescription drugs, with a large number of purchases probably at the wholesale level. Based on the results from a self-administered electronic questionnaire, higher-educated males and higher-income consumers were more likely to obtain medications online. This may be attributed to the fact that consumers tend to investigate the values and risks involved when buying medication online.

### 4.4. Consumer Safety

Consumer safety was discussed in most of the reviewed studies. Purchasing drugs online can be very risky and most consumers are unaware of the consequences. This is even more important when the online pharmacies are not recommended or illegal. Marketing strategies also contribute to the high demand for drugs. Apart from the advertisement of the advantages such as cheaper prices, other factors such as confidentiality and the desire to avoid physicians contribute to the increasing demand for online drugs. This is prevalent especially in countries other than the United States. In addition, the privacy issue has prompted consumers to believe that their data are protected. Hence, they rarely hesitate to reconsider the confidentiality issue. However, consumers are unaware that online pharmacies have a high potential to assist data-fishing or fraudulence when products are not delivered. These events will eventually lead to security issues.

### 4.5. Limitations and Future Research

The limitations of this study are well acknowledged. For example, most of the articles did not discuss the time existence and privacy policy of online pharmacies; thus information on these aspects was not included in this study. Additionally, articles written in languages other than English were not considered in this review. However, the papers from the search results were compared and analysed in detail using different techniques before selecting the eligible studies. These procedures enhanced the strength of this study.

### 4.6. Contributions/ Practical Implications

Half of the drugs sold online are shown to be of low quality, whereas an equal proportion was within the standard limits. In order to ensure consumer safety and prevent issues relating to undesirable complications, the quality of drugs needs to be taken more seriously. Additionally, more studies should be conducted in the future on the quality of prescription drugs.

As for the consumer characteristics, it was revealed that a very small number of consumers, such as drug addicts or drug abusers, attempt to misuse the drugs bought online. However, some consumers use online pharmacies as a shortcut to purchase drugs upon self-diagnosing their illness. This practice was mainly to reduce doctor’s fees and disregard in visiting a certified clinic or hospital. However, the practice is dangerous since they do not have sufficient knowledge to self-diagnose or self-medicate. Consumers should be more vigilant, particularly in distinguishing between legal and illegal online pharmacies.

Furthermore, patients and consumers should be enlightened about their medication use. On that note, consumers could reassess the information when visiting online pharmacies. Other features such as the requirements for proper packaging and proper labelling should be equally evaluated by consumers when engaging in online drug purchase.

## 5. Conclusions

This study revealed that almost half of the online pharmacies were not adequately regulated and fraudulent issues were uncovered. Therefore, to address these issues, stricter regulations should be implemented by the government agencies together with frequent monitoring of the licensure system and pharmacy verification on every online pharmacy. This would reduce the number of illegal online pharmacies and fraudulent practices.

## Figures and Tables

**Table 1:** Characteristics of Online Pharmacies.

Reference	Characteristic											
	1	2	3	4	5	6	7	8	9	10	11	12
Alwon, 2014 [2]	29		82	49						X		
Bachhuber, 2012 [13]	X				X					X		
Bate, 2010 [14]						X		X		X		
Campbell, 2012 [15]						X		X		X		
Fitller, 2013 [1]	7	49	75	46		X	X			X	X	
Fittler, 2018 [8]			X							X		
Floyd, 2017 [16]										X		
Gallagher, 2010 [17]	7	51	100	59								
Gelatti, 2013 [18]				X	X				X			
Liang, 2012 [19]	8									X		
Kunz, 2010 [20]										X		
Kuzma, 2011 [21]										X		
Lagan, 2014 [22]	X	33		98	X		X	X		14		
Levaggi, 2009 [23]	19	67		44	X	X		X	X	X		X
Levaggi, 2012 [24]					X			X				
Mahé, 2009 [25]	X					X		X	X			
Mainous, 2009 [26]	X	64			X	X						
Monteith, 2018 [27]	12			X						X		
NABP, 2011 [28]	4	56		37		X				X		
Orizio, 2009 [29]	19	56		43	X	X	X					
Orizio, 2009b [30]		70		28								
Orizio, 2011 [9]	22	45			X	X	X		X	X		
Rahman, 2018 [31]	52									X		
Raine, 2009 [32]	17	41		43	X	X	X	X				
van de Ven, 2017 [33]									X	X		
Vida, 2016 [34]	6			18				X	X	X		
Wang, 2015 [35]				X						X		

Characteristics: 1. Prescription requirement (%); 2. Online questionnaire (%); 3. Contact details (%); 4. Geographical locations (%); 5. Delivery; 6. Drugs offered; 7. Drug Information (%); 8. Drug Prices; 9. Marketing strategy; 10. Quality (%); 11. Duration to access the websites; 12. Privacy Policy. X indicates that data were analysed but real figure was not reported in the original article.

**Table 2:** Quality of drugs purchased online.

Author, Year of Publication	Years of Data Collection	Types of Drug Ordered	Response Rate (Product Received/Number of Order)	Drug Purchased Characteristics
Bate, 2010 [14]	2009	Atorvastatin (Lipitor^®^), sildenafil (Viagra^®^), celecoxib (Celebrex^®^), esomeprazole (Nexium^®^), sertraline (Zoloft^®^)	Response rate not computable; 152 ordered drugs were received	Prescription requirement
Dean, 2010 [36]	Not declared	Dapoxetine	100% (1/1)	Prescription requirement
Gaudiano, 2017 [37]	2005–2011	Phosphodiesterase type 5 (PDE5) inhibitors, e.g., sildenafil, tadalafil, and vardenafil	Not stated	Prescription requirement
Gelatti, 2013 [18]	2010–2011	Fluoxetine	13/64 received (20%)	Prescription requirement
Lagan, 2014 [22]	2011	Isotretinoin	7/8 (87.5%)	Prescription requirement
Mainous, 2009 [26]	2008	Antibiotics	100% (1/1)	Prescription requirement
Rahman, 2018 [31]	2013	Omeprazole	Not stated	Prescription requirement
Wang, 2015 [35]	2009–2011	HIV medications	2027 ordered capsules or tablets were received	Prescription requirement

**Table 3:** Characteristics of consumers buying products from online pharmacies.

Reference	Year Conducted	Country	Study Respondents	Number of Respondents	Study Design	Percentage of Respondents Purchasing Prescription Drugs Online
Atkinson, 2009 [38]	2005	USA	General population	5586	CS (HINTS, 2005 survey)	13% (715/5586) bought medicines or vitamins
Bechara, 2010 [39]	2009	Argentina	Healthy young men	321	CS using questionnaire on the use of phosphodiesterase type 5 inhibitors	2.9% (2/321)
Cohen, 2010 [40]	2009	USA	Sample of general population aged 18–64 years	7192	CS (NHI Survey, 2009b)	6% of 7192
Cicero, 2012 [41]	Not declared	USA	White females ranging from mid- to late 40s		CS	NA
Festinger, 2016 [42]	2010–2011	USA	Adolescent and young adults	1107	Web-based CS & NSDUH	26/1107 (2.3%)
Harte, 2010 [43]	2006–2007	USA	Male college and university students	77	CS using online questionnaires on the use of phosphodiesterase type 5 inhibitors	12% (8/77) of users
Inciardi, 2009 [44]	Varies with source	USA	Drug abusers, students, street sex workers, and individuals in “club culture” scene		RADARS System, NSDUH, Delaware School Study, Miami street studies, and qualitative studies	1–6%
Inciardi, 2011 [45]	Varies with source	USA	Drug abusers, students, and young adults		RADARS System, NSDUH, MTF survey	0.5–3%
Ivanistkaya, 2010 [46]	2005–2008	USA	Healthy young adults in their 20s (graduate and undergraduate students)		CS online assessment-RSSA-Health	NA
Jena, 2011 [47]	2000–2007	USA	Internet users		Data obtained from the High Speed Internet Development database, Federal Communications Commission.	NA
Ma, 2018 [48]	2012	USA	Participants of 18 years and above		MEPS data	18%
Mazer, 2010 [49]	2007	USA	Sample of emergency department patients	1654	CS using questionnaires	5.4% (89/1654)
Novak, 2016 [50]	Not declared	Denmark, Germany, Great Britain, Spain and Sweden	Sample age range 12 to 49 years		National survey	14.40%
Rajamma, 2009 [51]	Not declared	USA	Sample of general population born 1946–1964		CS using online questionnaires to a sample from the consumer panel by Common Knowledge Research Services	NA
Schnetzler, 2010 [52]	2008	UK, Germany and Italy	Sexually active men		CS using online questionnaires on the use of phosphodiesterase type 5 inhibitors	32%
Wiedmann, 2010 [53]	2008	Germany	Sample of the general population		Face-to-face interviews	NA

CS, cross-sectional; MTF, HINTS, Health Information National Trends Survey; MEPS, Medical Expenditure Panel Survey; MTF, Monitoring the Future; NSDUH, National Survey of Drug Use and Health; RRSA-Health, Research Readiness Self-Assessment, Health Version; UK, United Kingdom; USA, United States of America; RADARS, Researched Abuse Diversion and Addiction-Related Surveillance. NA = not available.
